# Yoga in Cancer Care: A Bibliometric Analysis of Systematic Reviews

**DOI:** 10.7759/cureus.71829

**Published:** 2024-10-19

**Authors:** Selvaraj Giridharan, Soni Soumian, Nagaraj V Kumar

**Affiliations:** 1 Oncology, Tawam Hospital, Al Ain, ARE; 2 General Surgery, Tawam Hospital, Al Ain, ARE; 3 Emergency Department, Tawam hospital, Al Ain, ARE

**Keywords:** bibliometric analysis, cancer care, integrative oncology, systematic reviews, yoga interventions

## Abstract

Yoga has gained recognition as a complementary intervention for managing the physical and psychological challenges faced by patients with cancer. Systematic reviews of yoga interventions have provided valuable insights into their roles in cancer care. This bibliometric analysis aimed to map the trends, contributors, and thematic focus of systematic reviews on yoga interventions in oncology. A comprehensive search was conducted across major databases, including PubMed, Scopus, and Web of Science, to identify systematic reviews and meta-analyses focusing on yoga interventions in cancer patients. Studies published between the inception of each database and July 2024 were included. Key data, such as authorship, year of publication, cancer type, outcomes assessed, and geographical distribution, were extracted and analysed. A total of 42 systematic reviews and meta-analyses were included in the final analysis. Most studies have focused on psychological outcomes including quality of life, stress reduction, and fatigue management, with a predominant focus on breast cancer. The majority of the research was led by authors from China and the United States, reflecting a growing global interest in yoga as an integrative cancer therapy. This analysis highlights the increasing research interest in yoga for cancer care, particularly regarding psychological outcomes. Future research should focus on underrepresented cancer types and physiological outcomes, and expand studies to low- and middle-income countries to optimise yoga’s role in global cancer care.

## Introduction and background

Cancer remains a significant global health challenge, with millions of new cases being diagnosed annually [1]. The disease, along with its often intensive treatments, such as chemotherapy, radiation, and surgery, frequently results in a range of debilitating physical, psychological, and emotional side effects, substantially affecting patients' quality of life. These effects include chronic pain, fatigue, anxiety, depression, and sleep disturbances, which persist well beyond the active treatment phase [2,3]. Consequently, there is increasing interest in exploring complementary and alternative medicine (CAM) practices that not only support the physical recovery of cancer patients but also enhance their emotional and psychological well-being [4-6].

Among these CAM approaches, yoga has gained considerable attention over the past two decades because of its holistic benefits in the management of cancer-related symptoms. Originating in ancient India, yoga is a mind-body discipline that integrates physical postures (asanas), breath control (pranayama), and meditation (dhyana) to promote mental clarity, emotional equilibrium, and physical health [7]. Beyond its spiritual roots, yoga has been adapted as a therapeutic modality in modern healthcare, particularly in oncology, to address the multidimensional challenges faced by cancer patients [8-11]. Unlike other physical activities, yoga's emphasis on mindfulness and breath awareness renders it uniquely suited to cancer patients whose energy levels and physical capabilities may be compromised during and after treatment [12].

Numerous clinical studies have highlighted the efficacy of yoga in alleviating cancer-related symptoms, such as fatigue, chronic pain, insomnia, anxiety, and depression [13-15]. These benefits are not limited to psychological well-being. Yoga has also been associated with physiological improvements, including enhanced immune function, improved physical mobility, and reduced levels of inflammation markers [16]. Importantly, yoga interventions offer a noninvasive, cost-effective option that can be tailored to individual patient needs, making it accessible and adaptable across diverse populations and stages of cancer treatment.

Systematic reviews and meta-analyses play a crucial role in synthesising this growing body of evidence, consolidating findings from multiple studies to provide a clearer and more reliable understanding of the effects of yoga on cancer patients [17]. These high-level reviews assess the quality and consistency of research and offer robust evidence to guide clinical decision-making. In recent years, an increasing number of systematic reviews have explored the effects of yoga interventions on various cancer-related outcomes, underscoring its potential to alleviate psychological distress, improve physical functioning, and support holistic recovery. Furthermore, the role of yoga in enhancing immune function and reducing inflammation is an emerging area of interest that reflects the broader trend towards integrating mind-body practices in conventional medical care [18].

Despite the growing volume of research, there is yet to be a comprehensive bibliometric analysis to systematically map the landscape of yoga research in cancer care. Bibliometric analysis is a powerful tool for evaluating the impact and trends within the literature, allowing researchers to identify key contributors, influential studies, and emerging themes [19]. By examining publication patterns, citation networks, and collaborative efforts, this study aimed to assess the evolution of systematic reviews of yoga interventions for cancer patients. This analysis will not only highlight leading authors, institutions, and journals but also map the geographical distribution of research efforts, providing a global perspective on the development of yoga research in oncology.

Given the rising interest in integrative oncology and the increasing recognition of the therapeutic potential of yoga, understanding these research trends is essential. By identifying research gaps and proposing directions for future studies, this bibliometric analysis aimed to contribute to the continued integration of yoga into comprehensive cancer care. Ultimately, this study seeks to optimise the role of yoga as a holistic, accessible intervention that can support both the physical and emotional recovery of cancer patients.

## Review

Methods

Study Design

This study employs a bibliometric analysis to evaluate the landscape of systematic reviews published on the effects of yoga on cancer patients. The analysis aims to map publication trends, identify key contributors, and explore the thematic evolution of research in this area. A comprehensive search was conducted to identify systematic reviews and meta-analyses focused on yoga interventions in cancer patients. The search was performed across major electronic databases: PubMed, Scopus, Web of Science, PROSPERO, and the Cochrane Library, from the earliest available records to July 2024. The search strategy included a combination of controlled vocabulary terms (e.g., MeSH) and free-text keywords. The search string used for each database included the following terms: ("Yoga" OR "Yoga Therapy") AND ("Cancer" OR "Oncology" OR "Neoplasm") AND ("Systematic Review" OR "Meta-Analysis") Searches were adapted to the specific syntax of each database. References were manually screened to ensure relevance and duplicate records were removed using EndNote citation management software. The search strategy followed Preferred Reporting Items for Systematic Reviews and Meta-Analyses (PRISMA) guidelines [20]. 

Eligibility Criteria

The inclusion and exclusion criteria were predefined to ensure the relevance of studies included in this bibliometric analysis. To qualify for inclusion, studies had to be published as systematic reviews or meta-analyses, specifically focusing on yoga interventions for cancer patients or survivors, regardless of the type of cancer. The reviews were required to be published in peer-reviewed journals and could include studies published from the inception of each database up until July 2024. Studies were excluded if they were narrative reviews, scoping reviews, or other non-systematic reviews. Additionally, reviews that focused on non-cancer populations or complementary therapies unrelated to yoga, as well as case reports, editorials, conference abstracts, or book chapters, were not considered for inclusion.

Data Extraction

Once the search process was completed, all identified articles were uploaded into EndNote for further processing. Titles and abstracts were screened by two independent reviewers to determine eligibility. In cases of disagreement, the reviewers resolved differences through discussion or consultation with a third reviewer. For each systematic review included in the analysis, key data points were extracted, including the author(s), publication year, journal name, and the country of the corresponding author. Additional details such as the institution(s) involved, the total number of citations, and relevant keywords were also documented. Furthermore, the extracted data included the review’s primary focus, which could be related to quality of life, psychological outcomes, or physiological outcomes. Other important information captured included the types of cancer addressed, the type of yoga intervention (if specified), and any reported funding sources.

Results

**Figure 1:**
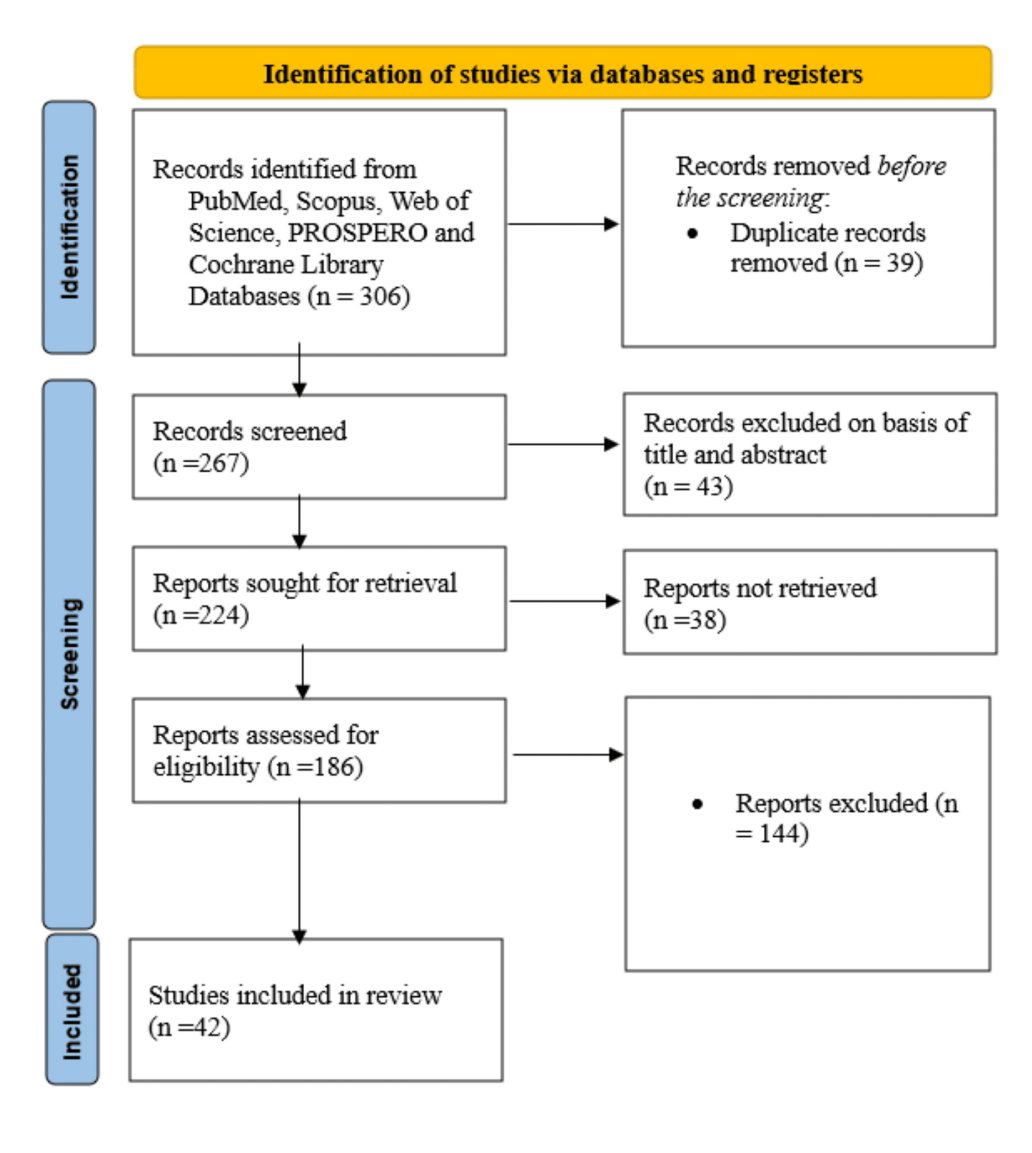
Summarized search strategy (Preferred Reporting Items for Systematic Reviews and Meta-Analyses flow diagram)

During the initial stage of the research selection process, 306 entries were identified across all evaluated databases, as shown in Figure 1. After removing 39 duplicates, 267 records remained for screening. Subsequently, 43 items were excluded due to the inaccessibility of their full content. Efforts were then made to obtain 224 reports, but 38 could not be retrieved for further examination (Figure 1).

The remaining 186 reports were assessed for eligibility, and 144 were excluded as they were either non-systematic reviews or off-topic. Ultimately, 42 systematic reviews and meta-analyses [21-62] were included in the final evaluation. Table 1 provides a detailed summary of the key characteristics of the included systematic reviews, including the study authors, year of publication, target cancer type, yoga intervention used, primary outcomes measured, and geographical distribution of the research (Table 1). 

**Table 1:** Summary of Systematic Reviews on Yoga Interventions in Cancer Care QoL: Quality of Life,  RCT: Randomized Controlled Trial, COB: Cochrane Risk of Bias tool, NA: Not Available, APTA: American Physical Therapy Association,  TNF-alpha: Tumor Necrosis Factor-alpha,  IL-6: Interleukin-6,  JBI: Joanna Briggs Institute,  PEDro:  The Physiotherapy Evidence Database, SIGN: Scottish Intercollegiate Guidelines Network,  CONSORT: The Consolidated Standards of Reporting Trials, GRADE: Grading of Recommendations Assessment, Development, and Evaluation, NOS: Newcastle-Ottawa Scale.

Author(s)	Year	Journal	Cancer Type	Review Focus	Number of studies	Sample Size	Quality tools used
Smith et al. [21]	2009	Psycho-Oncology	Various	Psychological adjustment	10	NA	Suydams measure - Likert scale
Chen et al. [22]	2011	Journal of Nursing and Healthcare Research	Various	Symptom relief, QoL	11	NA	NA
Lin et al. [23]	2011	Evidence-based Complementary and Alternative Medicine	Various	QoL, Psychological health, Physical health	10	NA	PEDro Score
Cramer et al. [24]	2012	Acta Oncologica	Breast	Fatigue	6	362	COB
Cramer et al. [25]	2012	BMC Cancer	Breast	QoL, Psychological health	12	742	COB
Zhang et al. [26]	2012	Journal of Alternative and Complementary Medicine	Breast	QoL, Psychological health	6	382	COB
Buffart et al. [27]	2012	BMC Cancer	Breast, Lymphoma	Physical, Psychosocial benefits	13	NA	Verhagens Delphi list
Harder et al. [28]	2012	Supportive Care in Cancer	Breast	Physical and psychological health	18	760	PEDro Score
Mustian et al. [29]	2013	Eur Med J Oncology	Various	Insomnia	NA	NA	NA
Sadja et al. [30]	2013	Explore	Various	Fatigue	10	583	COB
Sharma et al. [31]	2013	Journal of Alternative and Complementary Medicine	Various	Psychological and physical health	13	NA	NA
Sharma et al. [32]	2016	Journal of Cancer Research and Clinical Oncology	Breast	QoL, Physical health	23	NA	NA
Cramer et al. [33]	2017	Cochrane Database of Systematic Reviews	Breast	QoL, Psychological outcomes	24	2166	COB
Pan et al. [34]	2017	Asia-Pacific Journal of Clinical Oncology	Breast	Physical and psychological benefits	16	930	COB
Galliford et al. [35]	2017	Journal of Medical radiation sciences	Breast	Physical and Psychosocial QoL	38	NA	SIGN Methodology checklists
El-Hashimi et al. [36]	2019	Journal of Evidence-Based Integrative Medicine	Breast	QoL	8	545	NA
Wei et al. [37]	2019	Complementary Therapies in Clinical Practice	Breast	Lymphedema	5	NA	COB
Dong et al. [38]	2019	Breast Cancer Research and Treatment	Breast	Fatigue	17	2183	COB
Tang et al. [39]	2019	Sleep Medicine Reviews	Various	Sleep disturbance	25	1918	NA
O’Neill et al. [40]	2020	Integrative Cancer Therapies	Breast	Fatigue, QoL	24	NA	COB
Wanchai et al. [41]	2020	Journal of Health Research	Breast	Lymphedema	5	NA	NA
Baydoun et al. [42]	2020	Current Oncology Reports	Various	Cognitive function	10	NA	NA
Yi et al. [43]	2021	Annals of Palliative Medicine	Breast	QoL, Psychological and physical health	7	693	COB
Armer et al. [44]	2021	JNCI Cancer Spectrum	Various	Fatigue	29	1828	CONSORT score
Saraswathi et al. [45]	2021	International Journal of Yoga	Breast	Lymphedema	7	NA	COB
Song et al. [46]	2021	Oncology Nursing Forum	Various	Fatigue	16	NA	JBI Guidelines
Hseuh et al. [47]	2021	Breast Cancer	Breast	QoL	26	269	NA
Gonzalez et al. [48]	2021	Psycho-Oncology	Various	Depression, Anxiety	42	NA	COB
Selvan et al. [49]	2022	Complementary Therapies in Clinical Practice	Breast	Fatigue, nausea, anxiety, sleep	28	NA	NA
Nair et al. [50]	2023	Indian Journal of Palliative Care	Breast	QoL	17	2182	CASP
Zhu et al. [51]	2022	Frontiers in Oncology	Breast	Sleep quality	12	782	COB
Kanmodi et al. [52]	2023	Health Science Reports	Oral cancer	Stress, anxiety, and cognitive functioning	2	226	JBI guidelines
Price et al. [53]	2023	Complementary Therapies in Clinical Practice	Various	Women’s experiences with yoga after cancer diagnosis	24	490	NA
Levenhagen et al. [54]	2023	Rehabilitation Oncology	Breast	Lymphedema	9	NA	APTA
Ma et al. [55]	2023	Cancer Nursing	Various	Fatigue, QoL during chemotherapy	14	NA	COB
Kaje et al. [56]	2023	Indian Journal of Palliative Care	Breast	Inflammatory biomarkers, TNF-a, IL-6, Cortisol, Inflammatory markers	9	905	COB
Price et al. [57]	2023	Systematic Reviews	Various	Meta-synthesis of women’s experiences	24	490	NA
Garcia-Munoz et al. [58]	2024	Supportive Care in Cancer	Various	Fatigue, QoL	11	777	COB
Hou et al. [59]	2024	Medicine	Breast	Fatigue, sleep quality, and mood	18	1472	COB
Niu et al. [60]	2024	Asia-Pacific Journal of Oncology Nursing	Breast	Physical health, mental health, QoL	34	NA	COB
Qi et al. [61]	2024	European Journal of Oncology Nursing	Breast	Fatigue, pain, sleep disturbance	18	NA	COB
Boopalan et al. [62]	2024	Supportive care in cancer	Prostate	Fatigue, Urinary symptoms	5	129	COB, NOS

**Figure 2:**
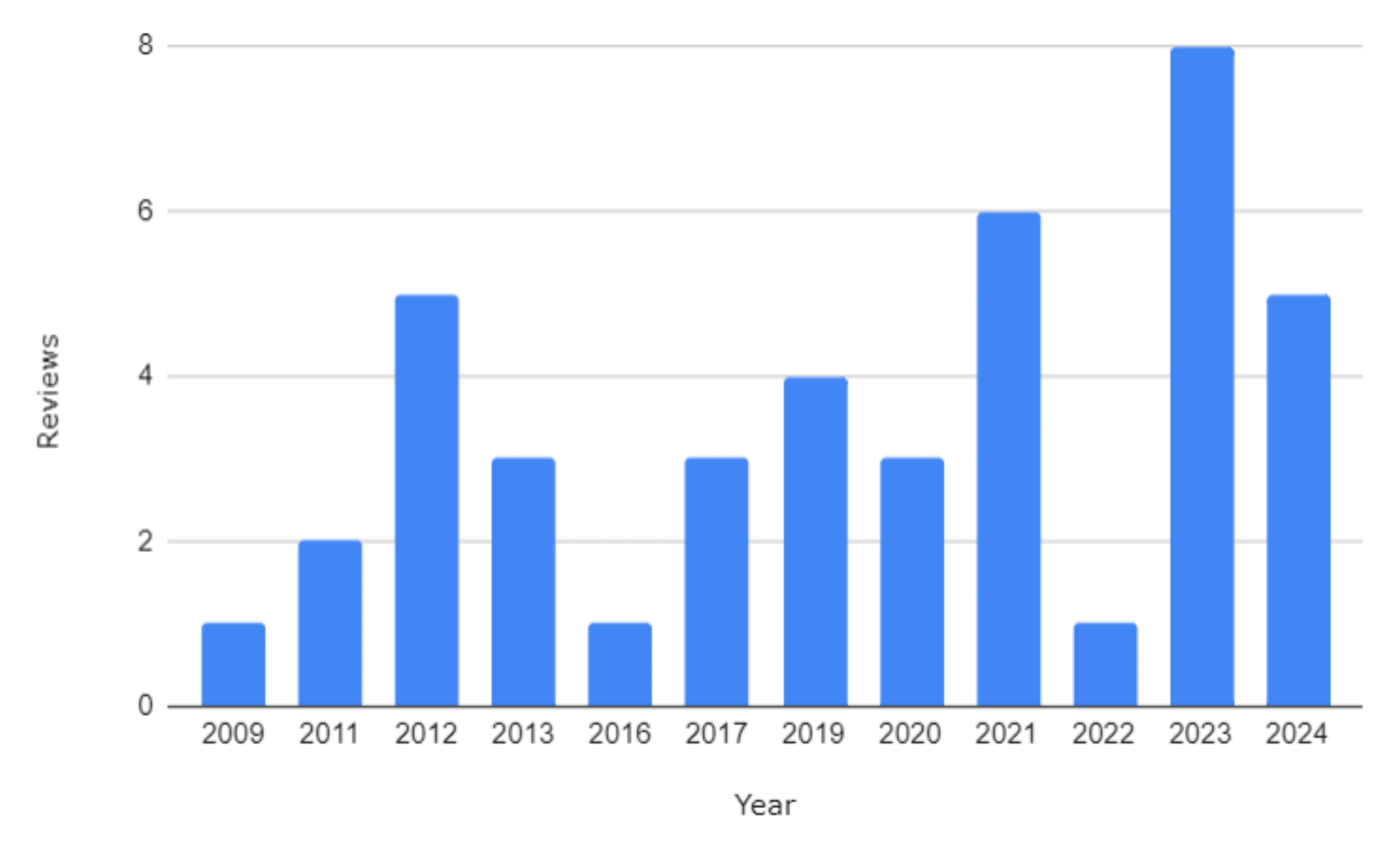
Annual Publication Trends of Systematic Reviews on Yoga Interventions in Cancer Care

The systematic reviews analysed in this bibliometric study span from 2009 to 2024, encompassing over a decade of research on yoga as a complementary therapy in cancer care. Throughout this period, there has been a notable increase in publications, particularly within the last five years, reflecting a growing interest in the potential benefits of yoga for cancer patients [36-63]. This upward trend suggests an intensification of research activity, aligning with the broader movement towards integrative oncology (Figure 2).

The journals with the highest number of publications were on *Complementary Therapies in Clinical Practice* and *Supportive Care in Cancer*, each with three reviews, this was followed by *BMC Cancer*, *Indian Journal of Palliative Care*, *Journal of Alternative and Complementary Medicine*, and *Psycho-Oncology*, each publishing two reviews. The diversity of publication venues reflects the multidisciplinary nature of yoga research in cancer care, encompassing contributions from general medical journals and oncology-specific publications.

Several key focus areas have emerged from these systematic reviews, with breast cancer as a central topic. This emphasis is likely due to the high prevalence of breast cancer and the well-documented benefits of yoga in symptom management in this patient population [24-26, 28, 32-38, 40, 41, 43, 46, 47, 49-51, 54, 56, 59, 60, 61]. Numerous reviews have also concentrated on the management of cancer-related fatigue, a common and debilitating symptom experienced by patients with cancer [24, 30, 38, 44, 46, 49, 55, 58, 59]. Other frequently explored themes include improvements in quality of life and the alleviation of psychological distress, such as anxiety and depression.

Additionally, some reviews have addressed sleep quality, cognitive function, and the management of lymphedema, particularly among breast cancer survivors [37, 41, 45, 54]. These diverse focus areas underscore yoga's potential to address a wide range of physical and psychological challenges in cancer care. 

**Figure 3:**
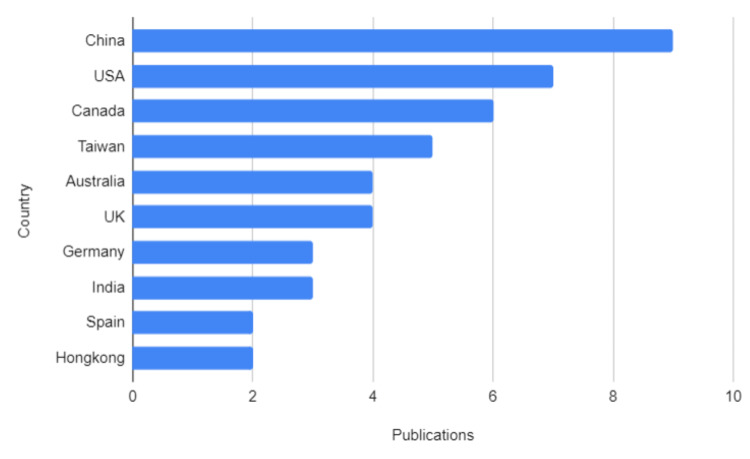
Geographical Distribution of Systematic Reviews on Yoga Interventions in Cancer Care

Geographically, the systematic reviews were conducted by researchers from a wide range of countries. China led with nine publications, followed by the United States with seven, Canada with six, and Taiwan with five. Other contributing countries included Australia, United Kingdom as well as Germany and India (Figure 3).

This distribution reflects the global interest in yoga as a complementary intervention for cancer patients and highlights its cross-cultural relevance in diverse healthcare systems. However, the relative underrepresentation of studies from low- and middle-income countries, despite their increasing cancer burden, suggests the need for expanded research efforts in these regions. Doing so could provide valuable insights and improve the accessibility of yoga as a therapeutic option in resource-limited settings.

Sample sizes varied across reviews; however, the majority included multiple randomised controlled trials (RCTs), providing robust data for meta-analyses. Larger sample sizes in the meta-analyses contributed to enhanced statistical power, resulting in more reliable conclusions regarding the efficacy of yoga interventions analyses [25, 27, 34, 36, 38, 39, 40, 43, 44, 46-48, 51, 53, 55, 57-60]. Through the inclusion of multiple studies, these reviews offer a comprehensive perspective on the effectiveness of yoga across diverse patient populations and clinical contexts.

The outcomes reported across the reviews were heterogeneous, reflecting the complexity of cancer care and the variability of yoga interventions. While numerous reviews have identified the positive effects of yoga on outcomes such as improved quality of life, reduced fatigue, and decreased psychological distress, others have reported no significant effects. This variability may be attributed to differences in yoga modalities, intervention duration and intensity, cancer types, or patient characteristics, underscoring the need for further research to elucidate these discrepancies. 

Several systematic reviews have compared yoga with other alternative interventions. For instance, one review examined yoga in comparison to walking for the management of sleep disturbances in patients with cancer [39]. Such comparative studies provide a valuable context for understanding the efficacy of yoga compared with other therapies. Future reviews could incorporate additional comparisons with standard care, exercise interventions, or other mind-body practices to better evaluate their comparative effectiveness. Some reviews provided details on specific yoga styles, such as Hatha or Iyengar yoga, while the majority did not specify the type of yoga utilised [38, 48, 49, 58]. The variability in yoga styles across studies likely contributes to the heterogeneity of outcomes. More consistent reporting of the specific styles and components of yoga employed in interventions could facilitate a refined understanding of how different forms of yoga impact cancer patients.

**Figure 4:**
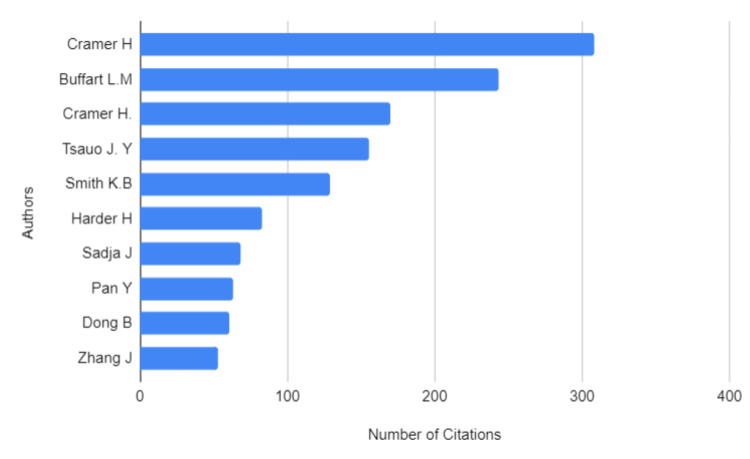
Author Citation Rankings in Systematic Reviews on Yoga Interventions in Cancer Care

The most frequently cited reviews were published between 2012 and 2013, suggesting that these early works played a foundational role in shaping the subsequent research (Figure 4).

These highly cited reviews have established the basis for yoga's recognition as a complementary therapy in cancer care, and their influence continues to inform newer studies. Citation patterns also revealed shifts in research priorities and methodological advancements over time as the field evolved. More recent reviews published in 2023-2024 tend to focus on specific outcomes or patient subgroups, reflecting a maturing field with more targeted research [52, 55, 56, 62]. This trend towards specialisation may be attributed to the growing body of high-quality primary studies now available, providing stronger evidence for particular aspects of yoga interventions in cancer care.

Methodological quality varied across the reviews. Some employed rigorous tools such as the Cochrane Risk of Bias tool to assess the quality of the included studies, while others did not. The heterogeneity of yoga interventions, outcome measures, and study designs posed challenges in synthesising evidence across reviews; however, these methodological considerations were crucial in assessing the strength and reliability of the findings.

The expanding body of systematic reviews and meta-analyses of yoga in cancer care provides valuable evidence for clinical practice and guideline development. While the results across reviews are mixed, the wide range of outcomes studied suggests that yoga may offer benefits across multiple domains of cancer care, including psychological well-being, physical functioning, and survivorship. Nevertheless, the mixed results also highlight the necessity for personalised approaches when recommending yoga to patients with cancer. 

There is a need for large-scale, high-quality RCTs to address the remaining uncertainties and refine the evidence base. Future reviews could explore the optimal timing, duration, and intensity of yoga interventions for different cancer populations, as well as the potential mechanisms of action, such as inflammatory markers or neurophysiological changes. This will enhance our understanding of how yoga functions in cancer care and potentially expand its application in integrative oncology.

Discussion

This bibliometric analysis represents the first comprehensive examination of systematic reviews focusing exclusively on yoga interventions in cancer care. It offers a detailed overview of the evolution of research in this field by identifying key trends, contributors, and thematic areas. While previous bibliometric analyses, such as those by Wieland et al, provided insights into systematic reviews of yoga across a broad range of health conditions, this analysis narrows the focus specifically to oncology - an area where the integration of complementary therapies is gaining prominence [63]. This study highlights the growing body of research exploring yoga’s impact on cancer-related outcomes, showing a significant increase in publications over the past decade, particularly since 2012. Most of the research in this field is led by authors from China and the United States, reflecting the broader global trend toward integrative oncology, where non-pharmacological interventions are increasingly valued for improving the quality of life and alleviating the side effects of conventional cancer treatments.

Key Findings and Their Implications

One of the most notable findings is the dominance of reviews focusing on psychological outcomes, particularly quality of life, stress reduction, and fatigue management. These topics reflect the growing understanding of how mind-body practices like yoga can alleviate mental health issues such as anxiety and depression, which are common among cancer patients. Influential reviews, such as those by Cramer et al. and Buffart et al, have helped solidify yoga’s role in improving mental health outcomes, particularly in cancer survivors [24, 25, 27, 33]. The high citation counts of these reviews indicate their significant impact on guiding future research and clinical practice. These findings underscore the importance of integrating yoga into psycho-oncology programs to support patients' mental well-being, demonstrating how well-regarded studies can influence both the academic and clinical landscapes.

However, while psychological outcomes have been well-researched, a key gap in the literature is the focus on specific cancer types. The majority of systematic reviews predominantly focus on breast cancer, possibly due to the large number of breast cancer survivors and established support programs in this population. This has created a noticeable imbalance, as other cancer types, such as gastrointestinal, lung, and prostate cancers, remain underrepresented in the literature. Future research should address these gaps by expanding yoga interventions to underrepresented cancer types and exploring their impact across different stages of cancer treatment, from diagnosis to palliative care.

In addition, despite the focus on psychological outcomes, there is a relative lack of studies investigating the physiological effects of yoga, such as its impact on immune function, tumor progression, and inflammatory markers. Although a few reviews have started to explore these aspects, they remain relatively scarce in the existing literature [56]. The lack of systematic exploration into these physiological outcomes represents a significant gap, especially given the growing interest in lifestyle interventions that modulate biological processes in cancer patients. Future research should prioritize these physiological outcomes to expand yoga’s clinical applicability beyond symptom management.

Geographically, this analysis reveals that much of the recent research in this field is led by authors from China and the United States. China's growing presence in yoga and cancer research is likely driven by its increasing embrace of integrative medicine, coupled with a rising cancer burden and government support for health-related research. In the United States, the prominence of integrative oncology research has also contributed to the number of systematic reviews conducted. Both countries reflect a global interest in yoga as a complementary therapy for cancer care.

However, there remains a noticeable underrepresentation of research from low- and middle-income countries (LMICs), despite their growing cancer burden. Expanding research efforts in these regions is crucial, as yoga presents a cost-effective and scalable intervention that could be particularly beneficial in resource-limited settings. Doing so would also provide valuable insights into how yoga can be adapted for diverse healthcare systems worldwide.

Strengths and Limitations

This bibliometric analysis has several strengths. By systematically including multiple databases and conducting a thorough examination of research trends, collaboration networks, and key contributors, this study provides a comprehensive overview of the field. The in-depth analysis allows for a detailed exploration of the thematic development of yoga research in oncology, offering valuable insights into the evolving focus areas and the global reach of the literature. This approach enhances the understanding of how yoga interventions have been integrated into cancer care and highlights emerging trends in research priorities across different regions and institutions.

There are also some limitations. Relying on citation counts as a measure of influence may not fully capture the quality or clinical significance of individual reviews. Studies with higher visibility might attract more citations while emerging or niche topics could be underrepresented. Additionally, the analysis primarily focused on English-language reviews, which may have excluded research contributions from non-English-speaking regions where yoga is widely practiced, such as parts of Asia.

Future Directions

This bibliometric analysis highlights several key areas for future research on yoga intervention in cancer care. One notable gap is the limited representation of studies from low- and middle-income countries (LMICs) where yoga could serve as a cost-effective therapy. Future studies should design culturally relevant interventions with cross-collaboration between high-income countries and LMICs to establish global best practices. While psychological benefits, such as stress and anxiety reduction, are well documented, further research is needed on the physiological effects of yoga, particularly its impact on immune function and inflammation.

Exploring the biological mechanisms of yoga could extend its therapeutic role beyond that of symptom management. The rise of digital health presents another opportunity, as virtual yoga platforms could improve access for cancer patients who face barriers to in-person participation. Investigating the effectiveness of virtual yoga programs could pave the way for broader adoption of cancer care protocols. Finally, more research should focus on underrepresented cancer populations, such as those with gastrointestinal or lung cancers, to address the current imbalance in the literature, which is predominantly focused on patients with breast cancer.

## Conclusions

This bibliometric analysis revealed the growing prevalence of systematic reviews on yoga interventions for cancer patients, primarily focusing on psychological outcomes. While mental health benefits are well established, additional research is needed to clarify the physiological effects of yoga on immune function, tumour progression, and inflammatory markers. The emphasis in the literature on breast cancer has led to the underrepresentation of other malignancies. Expanding yoga interventions to these less-studied populations will provide a more comprehensive understanding of the role of yoga in various cancer types and treatment stages. Increasing research in low- and middle-income countries, where yoga could be a cost-effective and accessible intervention, is essential to integrate yoga into global cancer care. Future research should explore yoga's physiological mechanisms and adapt interventions to a wider range of cancer types and treatment settings, refining yoga's role in comprehensive cancer care to meet the diverse needs of cancer patients globally.
